# Deletion of ELOVL6 blocks the synthesis of oleic acid but does not prevent the development of fatty liver or insulin resistance[S]

**DOI:** 10.1194/jlr.M054353

**Published:** 2014-12

**Authors:** Young-Ah Moon, Courtney R. Ochoa, Matthew A. Mitsche, Robert E. Hammer, Jay D. Horton

**Affiliations:** * Department of Molecular Genetics, University of Texas Southwestern Medical Center, Dallas, TX 75390-9046; † Department of Biochemistry, University of Texas Southwestern Medical Center, Dallas, TX 75390-9046; § Department of Internal Medicine, University of Texas Southwestern Medical Center, Dallas, TX 75390-9046

**Keywords:** fatty acid elongation, palmitic acid, sterol-regulatory element binding proteins

## Abstract

Elongation of very long chain fatty acid-like family member 6 (ELOVL6) is a fatty acyl elongase that performs the initial and rate-limiting condensing reaction required for microsomal elongation of long-chain fatty acids. Our previous in vitro studies suggested that ELOVL6 elongated long-chain saturated fatty acids and monounsaturated fatty acids with chain lengths of 12 to 16 carbons. Here, we describe the generation and phenotypic characterization of *Elovl6^−/−^* mice. As predicted from the in vitro studies, livers from *Elovl6^−/−^* mice accumulated palmitic (C16:0) and palmitoleic (C16:1, *n-*7) fatty acids and contained significantly less stearic (C18:0) and oleic (C18:1, *n-*9) acids, confirming that ELOVL6 is the only enzyme capable of elongating palmitate (C16:0). Unexpectedly, *Elovl6^−/−^* mice produced vaccenic acid (C18:1, *n-*7), the elongated product of palmitoleate (C16:1, *n-*7), suggesting that palmitoleate (C16:1, *n-*7) to vaccenate (C18:1, *n-*7) elongation was not specific to ELOVL6. The only detected consequence of deleting *Elovl6^−/−^* in mice was that their livers accumulated significantly more triglycerides than wild-type mice when fed a fat-free/high-carbohydrate diet. When mice were fed a high-fat diet or ELOVL6 was deleted in *ob/ob* mice, the absence of ELOVL6 did not alter the development of obesity, fatty liver, hyperglycemia, or hyperinsulinemia. Combined, these results suggest that palmitoleic (C16:1, *n-*7) and vaccenic (C18:1, *n-*7) acids can largely replace the roles of oleic acid (C18:1, *n-*9) in vivo and that the deletion of ELOVL6 does not protect mice from the development of hepatic steatosis or insulin resistance.

The major products of de novo fatty acid synthesis are saturated and monounsaturated fatty acids with chain lengths of 16 to 18 carbons (1, 2). Palmitate (C16:0) is synthesized from acetyl-CoA and malonyl-CoA through a series of reactions mediated by acetyl-CoA carboxylase (ACC) and fatty acid synthase (FAS) (3). Two carbons are added to palmitate (C16:0) through a series of microsomal fatty acid elongation enzymes to generate stearate (C18:0). In rodents, it is estimated that 90% of the endogenously synthesized stearate (C18:0) is produced from palmitate (C16:0) through the microsomal fatty acid elongation reactions (4). Palmitate (C16:0) and stearate (C18:0) are subsequently desaturated by a microsomal Δ9 desaturase, stearoyl-CoA desaturase (SCD), and converted to palmitoleate (C16:1, *n-*7) and oleate (C18:1, *n-*9), respectively (5, 6).

The enzymes required for the synthesis of fatty acids are regulated by the sterol-regulatory element binding protein (SREBP) family of transcription factors (7). Overexpression of the SREBP-1a or SREBP-1c isoforms in livers of mice resulted in fatty livers as a result of the transcriptional activation of ATP citrate lyase (ACL), ACC1, ACC2, FAS, and SCD1, which led to higher rates of de novo fatty acid synthesis and triglyceride accumulation (8, 9). The fatty acids that accumulated in the livers of the transgenic mice were enriched with oleic acid (C18:1, *n-*9) (10).

Elongation of very-long-chain fatty acid (ELOVL) 6, previously named long-chain fatty acyl-CoA elongase, was initially identified as a gene highly induced in livers of the SREBP transgenic mice (11, 12). ELOVL6 is an integral membrane protein with five predicted transmembrane domains and contains a histidine-rich motif (HXXHH), conserved sequences shared by yeast and other mammalian ELOVLs (11, 13–17). The ELOVL6 mRNA is expressed ubiquitously but is most highly expressed in white adipose tissue, brown adipose tissue, and liver, where rates of fatty acid synthesis are highest.

In vitro, fatty acid elongation assays using microsomal proteins prepared from HEK-293 cells that overexpressed ELOVL6 demonstrated that ELOVL6 mediated the initial rate-limiting condensation reaction of fatty acyl-CoA and malonyl-CoA, and its activity was restricted to long-chain saturated and monounsaturated fatty acyl-CoAs (C12-C16) (11, 18). The identification of *Elovl6* as a gene highly induced by SREBPs explained why livers of SREBP transgenic mice accumulated oleic acid (C18:1, *n-*9) (10). Like other SREBP-1-regulated genes in the fatty acid biosynthesis pathway, the mRNA expression of ELOVL6 was reduced in livers of fasted mice and increased more than 20-fold in livers of mice that were fasted and refed a high-carbohydrate/fat-free diet (18).

In the present study, we generated *Elovl6^−/−^* mice to determine the in vivo function of ELOVL6. We hypothesized that *Elovl6^−/−^* mice would have a defect in C16 fatty acid elongation, resulting in reduced oleate (C18:1, *n-*9) and accumulation of palmitate (C16:0) or palmitoleate (C16:1, *n-*7), which was found. Unexpectedly, we did not find significant alterations in overall fatty acid metabolism or in whole body energy expenditure, possibly due to the synthesis of vaccenic acid (C18:1, *n-*7).

## EXPERIMENTAL PROCEDURES

### Materials and general methods

Plasma glucose and insulin were measured using an Autokit Glucose CII kit from Wako Chemicals USA Inc. and an Ultra Sensitive Rat Insulin ELISA Kit from Crystal Chem Inc., respectively. Plasma free fatty acids were measured using a NEFA C kit from Wako Chemicals Inc. Cholesterol and triglycerides were measured in plasma and liver as described previously (8, 19). The fat-free/high-carbohydrate diet was purchased from MP Biomedicals (Cat. No. 960238), and mice were fed the diet for the days indicated in the figures. The high-fat/high-sucrose diet was purchased from Research Diets (Cat. No. D12451).

### Construction of *Elovl6* targeting vector

A mouse BAC clone that contained 100 kb of genomic DNA sequence of the mouse *Elovl6* gene was obtained from Incyte Genomics Inc., BAC Mouse II PCE library screening services. This clone covered 4 kb of the 5′ upstream region, exons 1–3, and a portion of intron 3. A gene-replacement targeting vector that deletes 1.2 kb of the promoter region and exons 1 and 2 (shown in supplementary Fig. IA) was constructed as follows. The short arm was amplified from the promoter region by PCR using the BAC clone as a template and the following primers: 5′ primer, 5′-TAGCCAAAGATGACCTTGAA-3′; and 3′ primer, 5-CTCGAG­CCTCTAAGATGTTCATTTCC-3′. The PCR product was digested with *Xho*I and ligated into the *Xho*I site of the pPollshort-neob­PA-HSVTK vector (20). The resulting plasmid was designated pElovl6KO-1. The long arm was amplified from the BAC clone by PCR using TaKaRa LA Taq^TM^ DNA polymerase (Takara) and the following primers: 5′ primer, 5′-GATATCCCC­ACTCA­GATTA­CTTTCCGGTTTCA-3′; and 3′ primer, 5′-GAACAGTGGGATATCATTTGTCCTCA-3′. The PCR product was digested with *Bam*HI and ligated into the *Bam*HI site of the pElovl6KO-1. This plasmid was designated pElovl6KO-2.

### Embryonic stem cell culture for disruption of *Elovl6*

Passage 10 SM-1 embryonic stem (ES) cells were electroporated with the *Elovl6* gene targeting vector pElovl6KO-2 as described previously (21). Recombined clones were screened by PCR using primers P4 (5′-TGTGCAGGTGAGCAGGTGCA-3′) from the promoter region of *Elovl6* and P3 (5′-GATTGGGAAGACAATAGCAGGCATGC-3′) from the 3′ untranslated region of the neocassette. The targeted clones were confirmed by Southern blot analysis using a 0.33 kb genomic DNA probe that contains a sequence within the *Elovl6* promoter region that is outside of the targeting vector (supplementary Fig. IA). The DNA probe was amplified by PCR from SM-1 genomic DNA using the following primers: 5′ primer, 5′-CTGGACTGATGACATCATTCCTGGT­TCT-3′; and 3′ primer, 5′-AAGGCAGAGACAAGATCGCTGCAA-3′. Southern blot analysis of genomic DNA digested with *Hind*III and hybridized with the ^32^P-labeled DNA probe was carried out as described previously (22). A 4.5 kb and a 6.6 kb band were detected for the wild-type and targeted allele, respectively (supplementary Fig. IC).

### Generation of *Elovl6^−/−^* mice

Two targeted ES cell clones were injected separately into C57BL/6J blastocysts, yielding chimeric males whose coat color (agouti) indicated a contribution of ES cells from 25% to 95%. Eight chimeric male mice with >90% agouti coat color were bred with C57BL/6J (Jackson Laboratory) female mice. The genotype of the offspring was identified by PCR of genomic DNA using the following primers: P1 (5′-GCTCTACTGTGCAATTTCCAGGATGG-3′); P2 (5′-GCTCCTAGCTCAGGGGCTCT-3′); and P3 (5′-GATTGGGAAGACAATAGCAGGCATGC-3′) (40 cycles, 94°C, 30 s; 60°C, 30 s; 65°C, 1 min). PCR amplification of the wild-type allele produced a product of 600 bp and amplification of the disrupted allele produced a product of 500 bp (supplementary Fig. IB). The genotype was confirmed by Southern blotting using the same methods described for the Southern blot analysis of the ES cells. Mice were housed in colony cages and maintained on a 12 h light/12 h dark cycle and fed Teklad Mouse/Rat Diet 7002 from Harlan Teklad Premier Laboratory Diets. *Elovl6^−/−^* mice in C57BL/6J genetic background were generated by breeding the mice with C57BL/6J female mice assisted with Marker-Assisted Accelerated Backcrossing (MAX-BAX, Charles River Laboratories) and confirmed ∼99% of C57BL/6J genetic locus. Female *Lep^ob^/^+^* mice (stock No. 000632) were purchased from Jackson laboratory and bred with *Elovl6^+/−^* males in C57BL/6J genetic background to generate *Elovl6^−/−^;ob/ob* mice. All animal studies were approved by the University of Texas Southwestern’s Institutional Animal Care and Use Committee.

### Northern blot analysis

Total RNA was isolated from livers of wild-type and *Elovl6^−/−^* mice and subjected to Northern blot analysis as described previously (8). The mouse *Elovl6* cDNA probe used in the Northern blot was amplified by PCR using pCMV-long-chain fatty acyl-CoA elongase (11) as the template with the following primers: 5′ primer, 5′-ATGAACATGTCAGTGTTGACT-3′; and 3′ primer, 5′-CTACTCAGCCTTCGTGGCTTTCTT-3′.

### ELOVL6 activity assay

ELOVL6 activity was measured in liver microsomes as described previously (11). Microsomes were prepared from wild-type and *Elovl6^−/−^* mice. [^14^C]palmitoyl-CoA (Amersham Biosciences Inc.) and malonyl-CoA or palmitoly-CoA, palmitoleoly-CoA, and arachidonoyl-CoA and [^14^C]malonyl-CoA (American Radiolabeled Chemicals Inc.) were added to the reaction mix and incubated with 50 μg of microsomal proteins. To separate radioactive palmitate (C16:0) and the elongated product, stearate (C18:0), the extracts of the elongation reactions were run through HPLC, and radioactivity in each fraction was measured.

### Lipid analyses

Wild-type and *Elovl6^−/−^* were fed a fat-free/high-carbohydrate diet (MP Biomedicals, Cat. No. 960238) for 3 days or 10 weeks. The fatty acid compositions were measured in ∼30 mg of the indicated tissues from individual mice. Fatty acids were extracted, and methyl esterified as described previously (10). Fatty acid methyl esters were separated by gas-liquid chromatography (GLC) using a Hewlett Packard 6890 Series GLC System (10). The identity of the fatty acid methyl esters was determined by comparing the retention times with fatty acid standards [Supelco 37 Component FAME Mix and PUFA-2, Animal Source (SUPELCO)]. Quantitative analyses of lipid classes in liver and the fatty acid composition were performed by Lipomics Technologies Inc.

### Immunoblot analyses

Membrane, nuclear, and cytosolic proteins were prepared from frozen liver as described previously (23). Equal amounts of proteins were subjected to SDS-PAGE using 6%, 8%, or 12% gels and transferred to Hybond ECL membrane (Amersham) or Trans-Blot Turbo Mini Nitrocelluose membrane (BioRad). Immunoblot analyses were performed using polyclonal anti-mouse SREBP-1 and SREBP-2 antibodies as described (8). To measure cytosolic lipogenic enzymes, antibodies that recognized ACL (Novus Biologicals), ACC1 (24), FAS (24), and SCD1 (Cell Signaling Technology Inc.) were used. Anti-cAMP response element binding protein (Cell Signaling Technology Inc.), anti-β-actin (Cell Signaling Technology Inc.), anti-transferrin receptor (Zymed), and anti-calnexin antibody (Enzo Life Sciences) were used for loading controls of hepatic nuclear, cytosolic, and membrane proteins, respectively.

### Quantitative real-time RT-PCR

Total RNA was prepared from livers of the indicated mice using RNA STAT-60 (TEL-TEST). RNA was treated with *DNase* I using DNA-free kit (Ambion Inc.), and first-strand cDNA was prepared using a TaqMan Reverse Transcription Reagents (Applied Biosystems). Quantitative real-time PCR was performed as described previously (25). The primers for real-time PCR were described previously (22, 25–27).

### Body fat analysis

Body fat mass was measured using a Minispec NMR analyzer (Bruker Analytik GmbH).

### Glucose and insulin tolerance tests

For glucose tolerance tests, mice were fasted for 16 h. Glucose solution (20%) was injected intraperitoneally at a dose of 2 g/kg body weight. Blood was collected from the retroorbital plexus under sedation with isoflurane, and plasma was separated. Plasma glucose and insulin levels were measured as described previously. For insulin tolerance tests, mice fed the high-fat/high-sucrose diet in ad libitum state were injected intraperitoneally with human regular insulin (Novolin, Novo Nordisk) at a dose of 1 U/kg body weight. Blood was collected from the tail vein, and glucose concentrations were measured using a glucometer (Bayer).

## RESULTS

The gene-targeting strategy used to disrupt *Elovl6* in mice is shown in supplementary Fig. IA. *Elovl6^−/−^* mice were generated by deleting 1.2 kb of the *Elovl6* promoter and the exons 1 and 2. Exon 2 contains the initiation ATG codon. Deletion of the targeted region in *Elovl6^−/−^* mice was confirmed by PCR and Southern blotting (supplementary Fig. IB, C). Northern blot analysis using RNA from livers of wild-type and *Elovl6^−/−^* mice hybridized with a ^32^P-labeled cDNA probe that covered the entire coding region of *Elovl6* failed to detect any ELOVL6 mRNA transcripts in *Elovl6^−/−^* mice (supplementary Fig. ID).

A total of 414 pups from breeding of *Elovl6^+/−^* mice in a mixed strain of 129S6/SvEv and C57BL/6J were examined, and the ratios of ^+/+^: ^+/−^: ^−/−^ were 1: 1.54: 0.5. Although there was partial embryonic or early neonatal lethality present, the surviving *Elovl6^−/−^* mice exhibited no visible growth defect. Adult *Elovl6^−/−^* mice fed a chow diet showed similar physiological parameters with wild-type mice (supplementary Table I). Body weight, liver weight to body weight ratios, liver triglyceride and cholesterol contents, plasma triglyceride and cholesterol levels, and plasma glucose and insulin levels of *Elovl6^−/−^* mice were not significantly different from those of wild-type mice. The gene expression levels of cholesterol and fatty acid synthetic enzymes were also unchanged. Body fat mass measured by NMR was similar between wild-type and *Elovl6^−/−^* mice (data not shown).

**Fig. 1.**
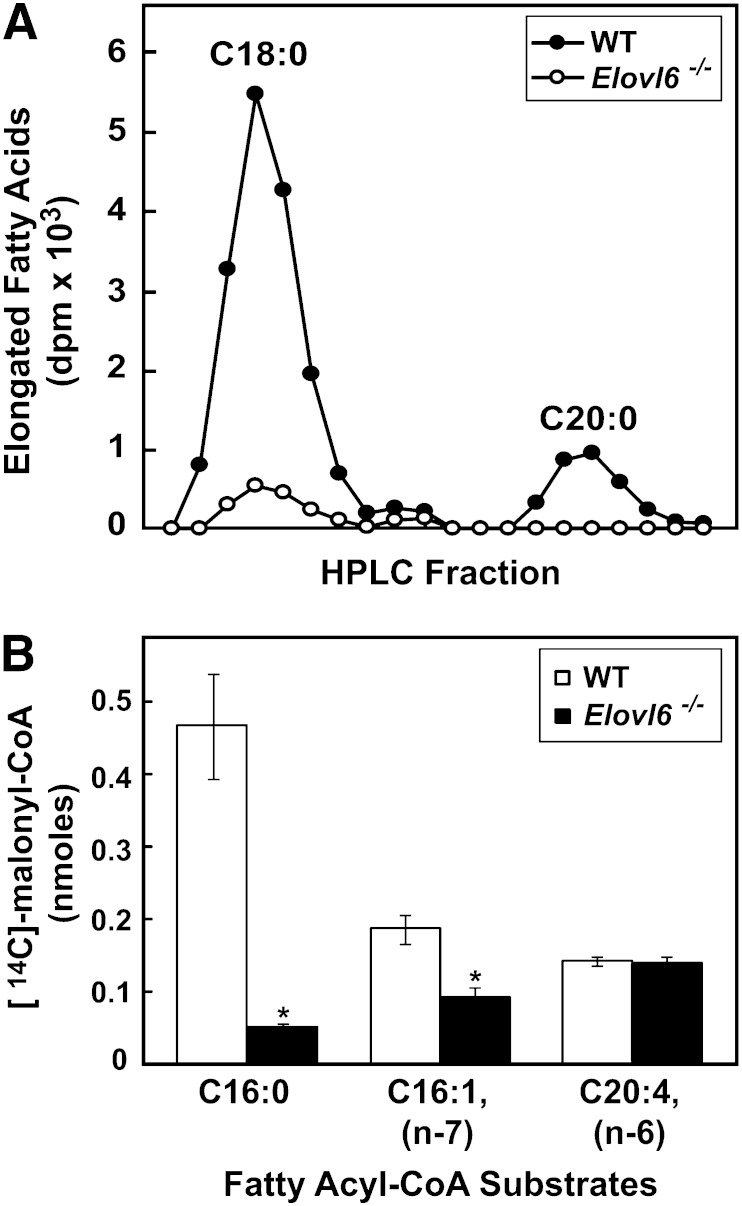
Elongation activity in livers of wild-type and *Elovl6^−/−^* mice. A: Microsomal proteins (50 μg) were incubated with a reaction mixture containing [^14^C]palmitoyl-CoA at 37°C for 10 min. The fatty acids in the reaction were extracted and separated by HPLC. Radioactivities in stearic acid (C18:0) and arachidic acid (C20:0) fractions are shown. B: Microsomal proteins (50 μg) were incubated with a reaction mixture containing the indicated fatty acyl-CoA and [^14^C]malonyl-CoA. The fatty acids in the reaction were extracted, and [^14^C] radioactivities were counted. The values are the mean ± SE of four mice.

To determine whether the deletion of *Elovl6* completely eliminated the elongation of palmitic (C16:0) to stearic (C18:0) acid, microsomes from livers of wild-type and *Elovl6^−/−^* mice fed the chow diet were incubated with [^14^C]palmitoyl-CoA, and the labeled products measured. Microsomes isolated from wild-type mice converted palmitate (C16:0) to stearate (C18:0), a percentage of which was further elongated to arachidate (C20:0), whereas essentially no stearate (C18:0) formation was measured in microsomes from *Elovl6^−/−^* mice (**Fig. 1A**).

To determine whether the deletion of *Elovl6* altered the overall fatty acid composition of tissues, fatty acids were extracted from livers of wild-type and *Elovl6^−/−^* mice fed a chow diet and analyzed by GLC. Inasmuch as stearic (C18:0) and oleic (C18:1, *n-*9) acids were the major fatty acid components of triglycerides in a standard chow diet, the *Elovl6^−/−^* mice did have slight increases of palmitic (C16:0), palmitoleic (C16:1, *n-*7), and vaccenic (C18:1, *n-*7) acids and small reductions of stearic (C18:0) and oleic (C18:1, *n-*9) acids (supplementary Table II).

To deplete fatty acids derived from the diet and to induce de novo fatty acid synthesis, mice were fed a fat-free/high-carbohydrate diet for 3 days. After 3 days on the fat-free/high-carbohydrate diet, the livers of *Elovl6^−/−^* mice accumulated 2-fold more triglycerides than wild-type mice (**Table 1**). However, additional physiological parameters including body weight, liver weight, liver cholesterol, plasma cholesterol and triglycerides, plasma free fatty acids, glucose, and insulin levels were not different from those of the wild-type controls.

**TABLE 1.** Phenotypic comparison of wild-type and *Elovl6^−/−^* mice

Parameter	WT	*Elovl6^−/−^*
Sex	Male	Male
Age (weeks)	15.3 ± 0.4	15.6 ± 0.6
Number of mice	10	10
Body weight (g)	27.0 ± 0.4	26.9 ± 1.0
Liver weight (g)	1.9 ± 0.0	1.9 ± 0.1
Liver cholesterol content (mg/g)	2.2 ± 0.1	2.6 ± 0.1
Liver triglyceride content (mg/g)	10.0 ± 1.8	27.8 ± 2.9*^a^*
Plasma cholesterol (mg/dl)	68 ± 6	85 ± 9
Plasma triglycerides (mg/dl)	160 ± 24	111 ± 17
Plasma glucose (mg/dl)	121 ± 5	106 ± 8
Plasma insulin (ng/ml)	2.8 ± 0.6	1.5 ± 0.5
Free fatty acids (mM)	0.48 ± 0.04	0.29 ± 0.08

Mice were fed a fat-free/high-carbohydrate diet for 3 days ad libitum. Each value represents the mean ± SE.

a Statistical significance of *P* < 0.01 analyzed by Student’s *t*-test.

To determine whether the deletion of *Elovl6* altered the composition of lipids, various lipid classes were extracted from liver, and the concentrations were measured (**Table 2**). *Elovl6^−/−^* livers had 2.3- and 2.8-fold higher concentrations of diglycerides and triglycerides, respectively. No differences in the concentrations of other lipid classes, including cholesteryl esters, free fatty acids, and phospholipids, were measured in *Elovl6^−/−^* livers.

**TABLE 2.** Major lipid classes in liver of wild-type and *Elovl6^−/−^* mice

Lipid Classes	WT	*Elovl6^−/−^*
Cholesteryl esters	3.6 ± 0.5	4.6 ± 0.8
Diglycerides	1.9 ± 0.5	4.4 ± 1.4*^a^*
Triglycerides	15.3 ± 7.3	41.9 ± 14.1*^a^*
Free fatty acids	7.3 ± 1.8	6.1 ± 1.4
Cardiolipin	2.2 ± 0.4	2.4 ± 0.1
Lysophosphatidylcholine	1.3 ± 0.2	1.4 ± 0.1
Phosphatidylcholine	13.8 ± 5.1	15.6 ± 0.9
Phosphatidylethanolamine	11.9 ± 0.8	10.8 ± 1.0
Phosphatidylserine	2.7 ± 0.8	2.9 ± 0.6
Sphingomyelin	1.3 ± 0.2	1.6 ± 0.3

Lipids were extracted from livers of wild-type and *Elovl6^−/−^* mice fed a fat-free/high-carbohydrate diet ad libitum for 3 days, and concentrations of the indicated lipid classes were measured. Each value represents the mean ± SE of the concentration (μmol/g tissue) of five mice.

a Statistical significance of *P* < 0.05 analyzed by Student’s *t*-test.

Next, the fatty acid compositions of the various lipid classes were measured (**Table 3**). Feeding the fat-free/high-carbohydrate diet for 3 days significantly changed the liver fatty acid composition. In all lipid classes, palmitoleic (C16:1, *n-*7), vaccenic (C18:1, *n-*7), and oleic (C18:1, *n-*9) acids were changed to the greatest extent. The relative amounts of palmitoleic (C16:1, *n-*7) and vaccenic (C18:1, *n-*7) acids in *Elovl6^−/−^* mice were 4.6- to 5.3-fold and 2.3- to 3.5-fold higher than those of wild-type mice, respectively. Conversely, the relative amounts of stearic (C18:0) and oleic (C18:1, *n-*9) acids in *Elovl6^−/−^* mice were 17% to ∼75% and 34% to ∼46% of those measured in wild-type mice, respectively. The relative amount of palmitic acid (C16:0) in triglycerides, diglycerides, free fatty acids, and the various phospholipids were 1.1- to ∼1.5-fold higher in *Elovl6^−/−^* mice. However, the total monounsaturated fatty acid content in livers of *Elovl6^−/−^* mice was not significantly different from that of wild-type mice owing to an increase in the amounts of palmitoleic (C16:1, *n-*7) and vaccenic (C18:1, *n-*7) acids. Feeding the fat-free diet for a longer period of time (10 weeks) resulted in exaggerated changes in the fatty acid composition of various tissues (supplementary Table III). While the changes in fatty acid composition were consistently detected, the levels of hepatic triglyceride concentrations in *Elovl6^−/−^* mice were variable across studies, although there was a tendency for higher hepatic triglyceride contents in *Elovl6^−/−^* mice.

**TABLE 3.** Fatty acid composition of major lipid classes from livers of wild-type and *Elovl6^−/−^* mice

Lipid Class	Elovl6	16:0	18:0	16:1, *n*-7	18:1, *n*-7	18:1, *n*-9	18:2, *n*-6	20:4, *n*-6	22:6, *n*-3	SFA	MUFA	*n*-7	*n*-9
CE	^+/+^	46.8	9.0	5.0	2.3	22.7	3.8	2.4	0.8	59.8	31.9	7.6	24.2
	^−/−^	43.3	2.9*^a^*	23.7*^a^*	6.4*^a^*	7.9*^a^*	2.8	1.6	0.4*^a^*	54.8	38.9	30.0*^a^*	8.7*^a^*
TAG	^+/+^	29.0	3.0	4.4	5.3	46.6	4.3	0.6	0.3	33.9	58.1	9.7	48.5
	^−/−^	38.3*^a^*	0.5*^a^*	22.9*^a^*	14.5*^a^*	16.5*^a^*	1.2	0.1	0.0	42.1*^b^*	55.2	37.4*^b^*	17.1*^b^*
DAG	^+/+^	29.3	4.4	4.1	3.7	41.3	6.6	2.4	0.3	36.7	50.7	7.8	43.1
	^−/−^	44.0*^b^*	1.3*^a^*	21.6*^a^*	9.0*^b^*	13.9*^b^*	1.9	0.9*^a^*	0.6*^a^*	48.4*^b^*	45.6	30.6*^b^*	14.8*^b^*
FFA	^+/+^	35.1	7.1	3.8	5.0	30.6	4.5	5.2	0.9	44.8	40.7	8.7	32.2
	^−/−^	41.3*^b^*	5.3	17.6*^b^*	11.3*^b^*	10.7*^b^*	3.5	3.2*^b^*	0.6	49.8*^a^*	40.6	28.9*^b^*	11.6*^b^*
PC	^+/+^	38.2	7.0	2.7	3.7	24.8	6.7	8.3	4.1	45.8	31.6	6.5	25.9
	^−/−^	41.6	2.9*^b^*	13.9*^b^*	13.0*^b^*	11.3*^b^*	4.2	6.1	3.2	45.1	38.4	26.9*^b^*	11.9*^b^*

CE, cholesteryl ester; DAG, diacylglycerols; PC, phosphatidylcholine; SFA, saturated fatty acid; TAG, triacylglycerols. Lipids were extracted from liver of wild-type and *Elovl6^−/−^* mice fed a fat-free/high-carbohydrate diet ad libitum for 3 days, and fatty acid composition was analyzed. Each value represents the mean of the indicated fatty acids (mol %) from five mice.

a Statistical significance of *P* < 0.05 analyzed by Student’s *t*-test.

b Statistical significance of P < 0.01 analyzed by Student’s *t*-test.

Although palmitoleic acid (C16:1, *n-*7) is a substrate for ELOVL6 in vitro (18), the elongated product of palmitoleic acid (C16:1, *n-*7), vaccenic acid (C18:1, *n-*7), was increased in *Elovl6^−/−^* livers. To determine whether the elongation of palmitoleic acid (C16:1, *n-*7) occurred in *Elovl6^−/−^* mice, wild-type and *Elovl6^−/−^* mice were fed the fat-free/high-carbohydrate diet for 1 week, and hepatic fatty acid elongation activities were measured in vitro using microsomes isolated from livers. As expected, the ability to elongate palmitate (C16:0) to stearate (C18:0) was reduced by >90% in microsomes from *Elovl6^−/−^* mice; however, the elongation of palmitoleate (C16:1, *n-*7) to vaccenate (C18:1, *n-*7) was reduced by only 50% (Fig. 1B). This result suggested that the elongation of palmitoleate (C16:1, *n-*7) was not solely dependent on ELOVL6, whereas ELOVL6 is required for the elongation of palmitate (C16:0) to stearate (C18:0). The increased vaccenic acid (C18:1, *n-*7) levels in *Elovl6^−/−^* livers despite a reduced ability to elongate palmitoleate (C16:1, *n-*7) suggested that other ELOVLs were capable of carrying out the condensation reaction for this fatty acid.

**Fig. 2.**
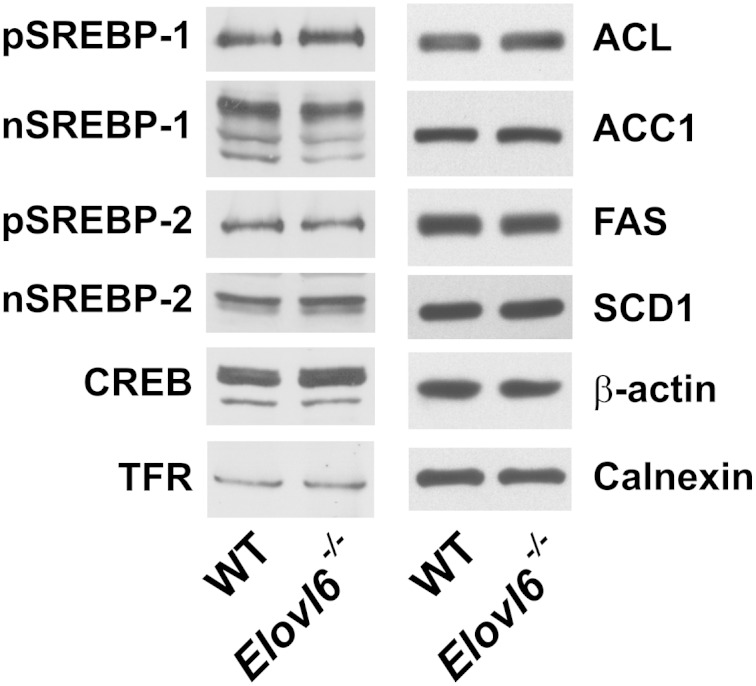
SREBP proteins and lipogenic enzyme levels in livers of wild-type and *Elovl6^−/−^* mice. Membrane, nuclear, and cytosolic proteins were prepared from livers from wild-type and *Elovl6^−/−^* mice presented in Table 1. Equal amount of proteins were pooled (30 μg for membrane and nuclear proteins and 10 μg for cytosolic proteins) and were subjected to SDS-PAGE and immunoblot analysis using primary antibodies for the indicated proteins as described under “Experimental Procedures.”

In *Drosophila* cells, palmitic acid (C16:0) suppresses SREBP cleavage and, thus, lipogenesis (28). To determine whether a similar regulatory mechanism exists in mice, we measured the amounts of precursor and nuclear forms of SREBP-1 and SREBP-2 by immunoblot analysis in livers of wild-type and knockout mice (**Fig. 2**). No significant changes in the precursor or the nuclear forms of SREBP-1 or SREBP-2 were measured in *Elovl6^−/−^* livers. Similarly, there were no significant changes in protein levels of enzymes involved in fatty acid synthesis such as ACL, ACC1, FAS, and SCD1 (Fig. 2). In addition, no compensatory increase in other ELOVLs expressed in liver (ELOVL1, ELOVL2, ELOVL3, and ELOVL5) was measured in *Elovl6^−/−^* livers (**Table 4**).

**TABLE 4.** mRNA expression levels of wild-type and *Elovl6^−/−^* mice

Genes	WT	*Elovl6^−/−^*
ApoB	1.0 ± 0.1	1.1 ± 0.1
Srebp-1c	1.0 ± 0.1	0.9 ± 0.1
Srebp-2	1.0 ± 0.1	1.1 ± 0.1
Fas	1.0 ± 0.1	0.8 ± 0.0
Scd1	1.0 ± 0.1	1.4 ± 0.1*^a^*
Hmgcs	1.0 ± 0.1	0.9 ± 0.1
Elovl1	1.0 ± 0.1	1.1 ± 0.1
Elovl2	1.0 ± 0.1	1.3 ± 0.1
Elovl3	1.0 ± 0.1	1.0 ± 0.1
Elovl5	1.0 ± 0.1	1.3 ± 0.1
Elovl6	1.0 ± 0.1	0.0 ± 0.0*^a^*
G3pat	1.0 ± 0.1	0.9 ± 0.1
Agpat2	1.0 ± 0.1	1.0 ± 0.1

Agpat, 1-acylglycerol-3-phosphate *O*-acyltransferase; G3pat, glycerol-3-phosphate acyltransferase; Hmgcs, HMG-CoA synthase. Mice were fed a fat-free/high-carbohydrate diet for 3 days ad libitum. Each value represents the mean ± SE of relative mRNA expression in the livers of 10 mice shown in Table 1. The values were normalized by cyclophilin, and the expression level of WT was designated as 1.0.

a Statistical significance of *P* < 0.01 of Student’s *t*-test.

**Fig. 3.**
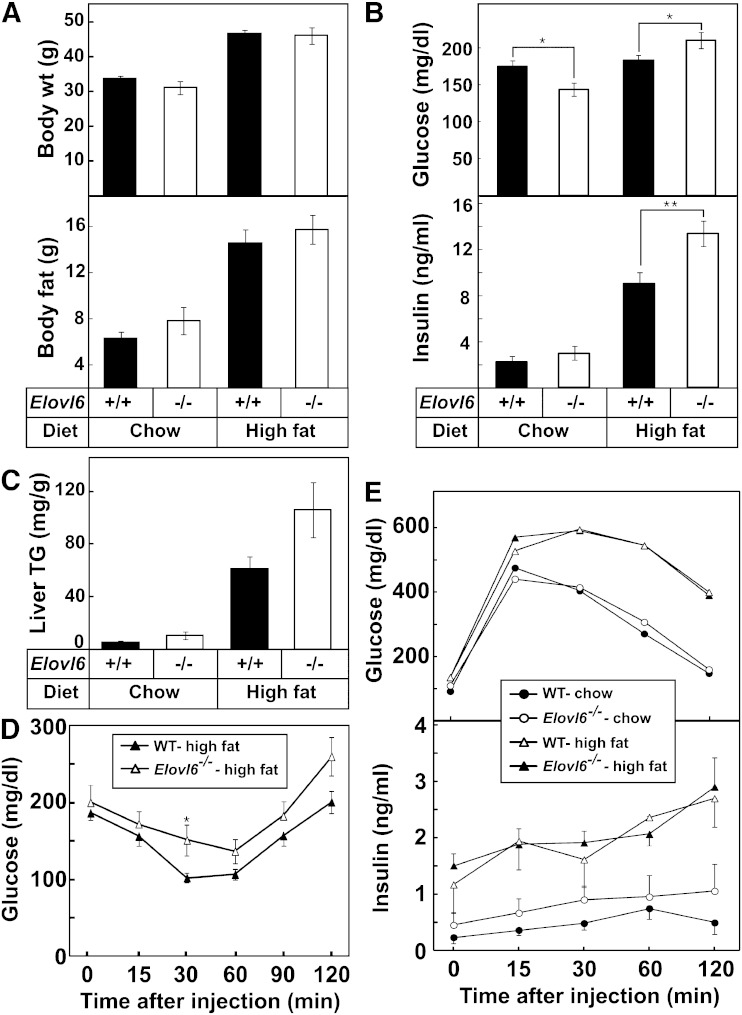
Metabolic phenotypes of wild-type and *Elovl6^−/−^* mice fed a high-fat/high-sucrose diet. Male mice in C57BL/6J genetic background were fed a high-fat/high-sucrose diet (45% kcal from fat) for 9 weeks. Body weight and fat mass (A), plasma glucose and insulin levels (B), and liver triglyceride concentrations of mice fed the chow diet and the high fat diet (C) are presented. The values are the mean ± SE of 8 mice (wild-type groups) and 5 mice (*Elovl6^−/−^* groups). D: Mice were injected with insulin (1 U/kg body weight) intraperitoneally, and plasma glucose concentrations were measured at the indicated time points. The values are the mean ± SE of 9 mice (wild-type) and 7 mice (*Elovl6^−/−^*). E: Mice were injected with glucose (2 g/kg body weight) intraperitoneally, and plasma glucose and insulin concentrations were measured at the indicated time points. The values are the mean ± SE of 8 mice (wild-type groups) and 5 mice (*Elovl6^−/−^* groups). * *P <* 0.05 and ** *P <* 0.01 (Student’s *t*-test).

ELOVL6 is required to synthesize oleic acid (C18:1, *n-*9) from palmitic acid (C16:0), which is the most abundant fatty acid in fatty livers of insulin-resistant mice (29). To determine whether the deletion of ELOVL6 could alter the development of insulin resistance, obesity, and fatty livers in response to caloric excess, wild-type and *Elovl6^−/−^* mice on the C57BL/6J background were fed a high-fat/high-sucrose diet (45% kcal from fat). At the end of 9 weeks, no significant differences were detected in body weight or body fat mass between wild-type and *Elovl6^−/−^* mice (**Fig. 3A**). Plasma glucose and insulin levels of *Elovl6^−/−^* mice were significantly higher than those of wild-type mice after high-fat feeding (Fig. 3B). Consistent with the increased plasma insulin level, both wild-type and *Elovl6^−/−^* mice exhibited pancreatic islet hyperplasia after high-fat feeding (supplementary Fig. II). In addition, *Elovl6^−/−^* mice accumulated more triglycerides and cholesterol in liver (Fig. 3C).

To determine whether whole body insulin sensitivity was affected, a glucose tolerance test and an insulin tolerance test were performed (Fig. 3D, E). *Elovl6^−/−^* mice developed the same degree of glucose intolerance as wild-type mice after feeding the high-fat/high-sucrose diet. Plasma insulin levels of *Elovl6^−/−^* mice during the glucose tolerance test were not different from those of wild-type mice. There was also no difference in the insulin tolerance tests between knockout and wild-type mice. Combined, the results suggest that the deletion of *Elovl6* does not protect the mice from the development of insulin resistance induced by a high-fat diet.

**Fig. 4.**
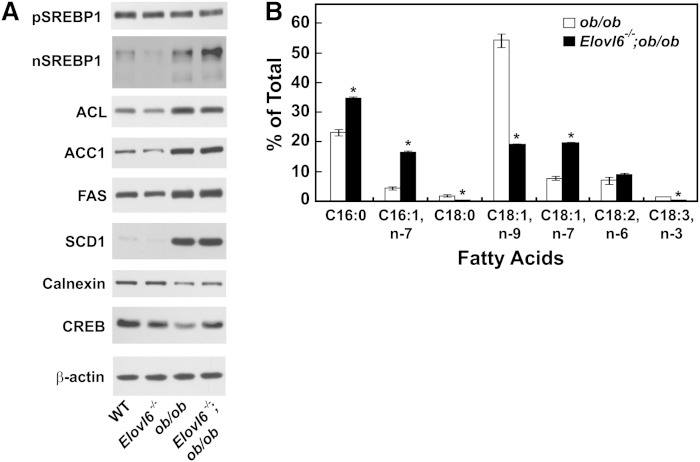
SREBP-1c protein, lipogenic enzymes, and fatty acid compositions of triglycerides of *ob/ob* and *Elovl6^−/−^;ob/ob* livers. A: Membrane, nuclear, and cytosolic proteins were prepared from livers from wild-type, *Elovl6^−/−^*, *ob/ob*, and *Elovl6^−/−^;ob/ob* mice presented in Table 5. Equal aliquots from samples were pooled (30 μg for membrane and nuclear proteins and 10 μg for cytosolic proteins) and were subjected to SDS-PAGE and immunoblot analysis using primary antibodies for the indicated proteins as described under “Experimental Procedures.” B: Liver triglycerides were extracted from livers of *ob/ob*, and *Elovl6^−/−^;ob/ob* mice, and fatty acids were methyl esterified. The fatty acid methyl esters were separated by GLC, and relative amounts of the indicated fatty acids compared with the total fatty acids were determined. The values are the mean ± SE of 5 mice.

To further confirm that the lack of ELOVL6 did not influence the development of obesity and diabetes, we generated mice that lacked ELOVL6 and leptin (*Elovl6^−/−^;ob/ob* mice). As shown in **Table 5**, the body weight, body fat mass, plasma cholesterol and triglyceride levels, plasma free fatty acids, insulin, and glucose levels of *ob/ob* and *Elovl6^−/−^;ob/ob* mice were not different. Unexpectedly, liver triglyceride contents were significantly higher in *Elovl6^−/−^;ob/ob* mice. Hyperinsulinemia in *ob/ob* mice drives activation of hepatic SREBP-1c, lipogenic genes, and thus de novo fatty acid synthesis, which results in the accumulation of triglycerides in liver that are enriched with oleic acid (C18:1, *n-*9) (29). *Elovl6^−/−^;ob/ob* mice showed similar activation of SREBP-1c and lipogenic enzymes as in *ob/ob* mice (**Fig. 4A**). The increased de novo fatty acid synthesis in the *ob/ob* background resulted in a dramatic change in the hepatic fatty acid composition of *Elovl6^−/−^* mice compared with that of wild-type controls (Fig. 4B). Although oleic acid (C18:1, *n-*9) levels in hepatic triglycerides of *Elovl6^−/−^;ob/ob* were decreased, the increase of palmitoleic (C16:1, *n-*7) and vaccenic (C18:1, *n-*7) acids resulted in similar amounts of total monounsaturated fatty acids.

**TABLE 5.** Phenotypic comparison of wild-type, *Elovl6^−/−^*, *ob/ob*, and *Elovl6^−/−^*; *ob/ob* mice

Parameter	WT	*Elovl6^−/−^*	*ob/ob*	*Elovl6^−/−^;ob/ob*
Number of mice	5	5	5	5
BW (g)	27.7 ± 1.2	23.8 ± 0.9*^a^*	47.7 ± 2.6	46.1 ± 3.4
Body fat mass (%)	12.1 ± 1.7	9.6 ± 0.7	46.7 ± 1.0	50.3 ± 1.9
LW (g)	1.4 ± 0.1	1.2 ± 0.0	4.0 ± 0.3	5.1 ± 0.4
LW/BW (%)	5.0 ± 0.3	5.2 ± 0.1	8.4 ± 0.6	11.1 ± 0.6*^a^*
Liver cholesterol (mg/g)	2.7 ± 0.1	2.4 ± 0.1	3.3 ± 0.2	4.7 ± 0.4*^a^*
Liver triglyceride (mg/g)	6.4 ± 1.0	4.6 ± 0.3	131 ± 5	209 ± 27*^a^*
Plasma cholesterol (mg/dl)	114 ± 8	91 ± 4*^a^*	178 ± 21	209 ± 25
Plasma triglycerides (mg/dl)	104 ± 23	133 ± 31	89 ± 9	105 ± 28
Plasma glucose (mg/dl)	234 ± 19	228 ± 16	427 ± 48	348 ± 19
Plasma insulin (ng/ml)	0.82 ± 0.16	1.06 ± 0.13	57 ± 14	60 ± 6
Plasma free fatty acids (mM)	0.84 ± 0.08	0.89 ± 0.04	0.67 ± 0.04	1.05 ± 0.12

Male mice, 12–13 weeks of age, were fed a chow diet ad libitum. Each value represents the mean ± SE.

a Statistical significance of *P* < 0.05 (Student’s *t*-test). BW, body weight; LW, liver weight.

## DISCUSSION

In this study, we generated *Elovl6^−/−^* mice to determine the in vivo function of ELOVL6 in the fatty acid synthesis pathway and physiological importance of ELOVL6 in lipid and glucose homeostasis. The main activity of ELOVL6 in vivo was the elongation of palmitate (C16:0) to stearate (C18:0). When animals were dependent on de novo fatty acid synthesis as a result of being fed a fat-free/high-carbohydrate diet or stimulated as in the *ob/ob* background, the deletion of *Elovl6* resulted in dramatic changes of tissue fatty acid composition, the reduction of cellular stearic (C18:0) and oleic (C18:1, *n-*9) acids and increased palmitic (C16:0), palmitoleic (C16:1, *n-*7), and vaccenic (C18:1, *n-*7) acids.

Consistent with previous in vitro fatty acid elongation assays, microsomes prepared from *Elovl6^−/−^* mice were largely unable to elongate palmitate to stearate, whereas they retained the ability to elongate palmitoleate albeit at a reduced rate. The combined results were lower concentrations of stearic (C18:0) and oleic (C18:1, *n-*9) acids with a concomitant increase in palmitic (C16:0) and palmitoleic (C16:1, *n-*7) acids (11, 18). Unexpectedly, *Elovl6^−/−^* mice synthesized vaccenic acid (C18:1, *n-*7) efficiently from the elongation of palmitoleate (C16:1, *n-*7), which led to a normal monounsaturated fatty acid level in tissues of ELOVL6-deficient mice.

One ELOVL capable of elongating palmitoleic (C16:1, *n-*7) to vaccenic (C18:1, *n-*7) acids is ELOVL5 (30). When ELOVL5 was overexpressed in mouse liver using adenovirus, an increase in vaccenic acid (C18:1, *n-*7) content was observed (31). However, the significant accumulation of palmitoleate (C16:1, *n-*7) in *Elovl6^−/−^* tissues suggests that palmitoleate elongation to vaccenate (C18:1, *n-*7) is not as efficient as the palmitate (C16:0) to stearate (C18:0). Despite significant changes in the tissue fatty acid profiles, *Elovl6^−/−^* mice did not manifest any significant changes in physiological parameters except for having a tendency for higher liver triglyceride contents.

Recently, several reports have shown potential roles for palmitoleic acid (C16:1, *n-*7) in the regulation of lipogenesis in mice (32, 33). Cao et al. (32) showed that palmitoleate (C16:1, *n-*7) generated from adipose tissue acted as a lipokine that could reduce the expression of genes involved in fatty acid synthesis in liver. In another study, dietary palmitoleate (C16:1, *n-*7) supplementation led to hepatic steatosis by inducing SREBP-1c and FAS mRNAs (33). Although *Elovl6^−/−^* mice fed the fat-free/high-carbohydrate diet and *Elovl6^−/−^;ob/ob* mice exhibited increased plasma palmitoleate (C16:1, *n-*7), which was likely released from adipose tissue, it did not affect hepatic gene expression or proteins of fatty acid synthesis in this animal model (Fig. 2, Fig. 4; supplementary Fig. III).

Mice chronically fed high-fat diets develop insulin resistance as they become obese. Matsuzaka et al. (34) also studied mice that lacked ELOVL6 and showed that the absence of ELOVL6 protected mice from developing hyperinsulinemia and hyperglycemia in a diet-induced obesity model and in leptin-deficient *ob/ob* mice. However, in stark contrast to their studies, the *Elovl6^−/−^* mice we generated were not protected from diet-induced insulin resistance or obesity. Both wild-type and *Elovl6^−/−^* mice manifested pancreatic islet hyperplasia that parallels the development of hyperinsulinemia. SREBP-1c protein was equally active in livers of *Elovl6^−/−^* mice as in the liver of wild-type mice as determined by measuring the active form of SREBP-1 protein and the mRNA expression levels of its target genes (data not shown). When *Elovl6^−/−^* mice were bred to *ob/ob* mice, an animal model of insulin resistance and obesity, the degree of obesity, fatty liver, hyperglycemia, or hyperinsulinemia were not different than in *ob/ob* mice.

Also in contrast to previous reports (34, 35), the concentration of vaccenic acid (C18:1, *n-*7) in livers of the *Elovl6^−/−^* mice we generated was elevated, which could be a compensatory response to replace C18 monounsaturated fatty acids. These results suggest that *Elovl6^−/−^* mice could synthesize other monounsaturated fatty acids such as palmitoleic (C16:1, *n-*7) and vaccenic (C18:1, *n-*7) acids efficiently, and these monounsaturated fatty acids might replace the roles of oleic acid (C18:1, *n-*9) in vivo. Currently, we cannot explain the reasons for the discrepancies in the two lines of ELOVL6 knockout mice other than possible differences in the animal housing environments and in the manner in which ELOVL6 was deleted.

Recently, inhibitors for mammalian ELOVL6 were developed, and the compounds were tested in diet-induced obesity animals and KKAy mice, a genetically obese and diabetic animal model. While the compounds effectively inhibited ELOVL6 activity and changed tissue fatty acid compositions, no improvement in insulin resistance was observed (36). These findings are consistent with the results we obtained in the ELOVL6 knockout mice.

Interestingly, livers from *Elovl6^−/−^* mice fed a fat-free/high-carbohydrate diet or a high-fat diet and *Elovl6^−/−^;ob/ob* mice actually accumulated more triglycerides than their respective controls. Using primary hepatocytes isolated from *Elovl6^−/−^* mice fed the fat-free/high-carbohydrate diet, rates of triglyceride synthesis, triglyceride secretion, and fatty acid oxidation were measured and failed to show differences between wild-type and *Elovl6^−/−^* mice (data not shown). Further studies will be needed to decipher the mechanism of how the changes in fatty acid composition shown in *Elovl6^−/−^* mice can affect hepatic triglyceride concentrations.

Palmitoleate (C16:1, *n-*7) and vaccenate (C18:1, *n-*7) possess a double bond in a different position (*n-*7) from oleate (C18:1, *n-*9), and this may alter the spatial arrangements of fatty acids in phospholipids and thereby fluidity. Future studies will determine whether the increase in these fatty acids alter additional aspects of lipid metabolism not investigated here. Nevertheless, the current studies suggest that the maintenance of monounsaturated fatty acids in the absence of ELOVL6 is sufficient to maintain normal cellular function and lipid metabolism under the conditions studied.

## Supplementary Material

Supplemental Data
