# Supplementary material for: Deletion of ELOVL6 blocks the synthesis of oleic acid but does not prevent the development of fatty liver or insulin resistance
Source: J Lipid Res. 2014 Dec;55(12):2597–605. doi: 10.1194/jlr.M054353 (PMC4242452; doi:10.1194/jlr.M054353)
Supplement: Supplemental Data [file supp_55_12_2597__index.html]

Deletion of ELOVL6 Blocks the Synthesis of Oleic Acid but does not Prevent the Development of Fatty Liver or Insulin Resistance — Deletion of ELOVL6 blocks the synthesis of oleic acid but does not prevent the development of fatty liver or insulin resistance — Supplemental Data 

# Deletion of ELOVL6 blocks the synthesis of oleic acid but does not prevent the development of fatty liver or insulin resistance

## Supplemental Data

**Files in this Data Supplement:**

- Supplemental data - Supplemental information, tables, and figures
