# Supplementary material for: Deletion of ELOVL6 blocks the synthesis of oleic acid but does not prevent the development of fatty liver or insulin resistance
Source: J Lipid Res. 2014 Dec;55(12):2597–605. doi: 10.1194/jlr.M054353 (PMC4242452; doi:10.1194/jlr.M054353)
Supplement: Supplemental Data [file supp_M054353_jlr.M054353-1.pdf]

Supplemental Data

Supplemental methods

Analysis of plasma free fatty acid compositions

Plasma free fatty acid compositions of *ob/ob* and *Elovl6*$^{-/-}$*;ob/ob* mice were measured using 100 μl of plasma by liquid chromatography-tandem mass spectroscopy analysis (LC-MS/MS). Fatty acids were extracted by the Bligh-Dyer's method (1) with an internal standard (^{13}C-16-palmitic acid). The organic phase of the extract was evaporated and resuspended in 300 μl of acetonitrile (ACN)/water (3:2). The fatty acids were separated by a Shimadzu LC20-AD HPLC with a phenyl-hexyl column (Phenomenex) under a linear gradient of ACN/water from 6:4 to 8:2. Fatty acids were detected using an ABSciex 4000 Q-trap MS/MS in negative mode with electrospray ionization. The peak area of each fatty acid was integrated using Analyst® software and normalized to the added ^{13}C-16-palmitic acid.

Immunofluorescent staining of pancreas

Pancreas was fixed in Bouin's solution and slides were prepared. Iimmunofluorescent staining was performed as described (2,3) using guinea pig anti-insulin (DAKO) and rabbit anti-glucagon (LINCO) as primary antibodies and goat anti-rabbit Ig FITC (Jackson Laboratories) and donkey anti-guinea pig Ig TRITC (Jackson Laboratories) as secondary antibodies.

References

1. Bligh, E. G., and Dyer, W. J. (1959) A rapid method of total lipid extraction and purification. *Canadian journal of biochemistry and physiology* **37**, 911-917

2. Orci, L., Baetens, D., Rufener, C., Amherdt, M., Ravazzola, M., Studer, P., Malaisse-Lagae, F., and Unger, R. H. (1976) Hypertrophy and hyperplasia of somatostatin-containing D-cells in diabetes. *Proc. Nat. Acad. Sci.U. S. A.* **73**, 1338-1342
3. Wang, Z. V., Mu, J., Schraw, T. D., Gautron, L., Elmquist, J. K., Zhang, B. B., Brownlee, M., and Scherer, P. E. (2008) PANIC-ATTAC: A Mouse Model for Inducible and Reversible β-Cell Ablation. *Diabetes* **57**, 2137-2148

Supplemental TABLE 1.

Phenotypic comparison of wild-type and *Elovl6*$^{-/-}$ mice

**Parameter**	**WT**	***Elovl6***$^{-/-}$
Age (weeks)	12.6 ± 0.7	12.7 ± 0.6
Number of mice	9	9
BW (g)	26.2 ± 1.0	27.5 ± 1.0
LW (g)	1.4 ± 0.1	1.8 ± 0.1*
LW/BW (%)	5.5 ± 0.4	6.5 ± 0.4
Liver cholesterol (mg/g)	2.3 ± 0.1	2.2 ± 0.1
Liver triglyceride (mg/g)	6.6 ± 0.7	8.0 ± 1.0
Plasma cholesterol (mg/dl)	100 ± 5	104 ± 6
Plasma triglycerides (mg/dl)	143 ± 18	171 ± 23
Plasma glucose (mg/dl)	223 ± 16	189 ± 10
Plasma insulin (ng/ml)	1.7 ± 0.3	2.4 ± 0.5
Free fatty acids (mM)	0.41 ± 0.03	0.31 ± 0.02

Male mice were fed a chow diet *ad libitum*. Each value represents the mean ± S.E.. * indicates $p < 0.05$ of Student *t*-test.

Supplemental TABLE 2.

Liver fatty acid composition of wild-type and *Elovl6*$^{-/-}$ mice

**Parameter**	**WT**	***Elovl6***$^{-/-}$
C16:0	31.0 ± 2.1	33.6 ± 0.7
C16:1, n-7	1.5 ± 0.2	2.9 ± 0.2**
C18:0	12.7 ± 0.6	10.6 ± 0.3*
C18:1, n-9	16.6 ± 1.8	14.2 ± 0.6
C18:1, n-7	2.3 ± 0.2	2.9 ± 0.1
C18:2, n-6	15.3 ± 0.5	16.6 ± 0.2
C20:4, n-6	14.9 ± 0.5	13.2 ± 0.7
C22:6, n-3	5.8 ± 0.3	6.2 ± 0.2

Mice were fed a chow diet *ad libitum*. Relative amounts of the indicated fatty acids compared to the total fatty acids in liver (%) are shown. Each value represents the mean ± S.E. of 4 animals. * and ** indicate $p < 0.05$, and $p < 0.01$ of Student *t*-test, respectively.

Supplemental TABLE 3.

Fatty acid composition of various tissues in wild-type and *Elovl6*$^{-/-}$ mice

	Elovl6	C16:0	C16:1	C18:0	C18:1, n-9	C18:1, n-7
Liver	+/+	21.2 ± 0.8	4.9 ± 0.2	4.6 ± 0.2	52.4 ± 1.1	8.2 ± 0.2
	-/-	**31.4 ± 0.6**	**17.6 ± 1.2**	**1.6 ± 0.2**	**19.9 ± 0.9**	**24.5 ± 0.7**
BAT	+/+	26.7 ± 0.4	8.8 ± 0.1	5.3 ± 0.1	49.5 ± 0.3	4.6 ± 0.1
	-/-	**37.3 ± 0.9**	**31.1 ± 0.4**	**0.5 ± 0.0**	**14.2 ± 0.4**	**10.3 ± 0.6**
WAT	+/+	24.0 ± 0.5	10.6 ± 0.4	3.3 ± 0.3	54.0 ± 0.5	4.6 ± 0.1
	-/-	**32.3 ± 1.1**	**31.4 ± 1.2**	**0.5 ± 0.0**	**18.5 ± 0.6**	**13.1 ± 0.8**
Muscle	+/+	21.7 ± 0.6	9.7 ± 0.5	4.1 ± 0.5	53.2 ± 1.2	6.0 ± 0.2
	-/-	**29.0 ± 0.7**	**28.9 ± 1.4**	**1.0 ± 0.2**	**20.3 ± 0.9**	**14.8 ± 0.7**
Brain	+/+	22.8 ± 0.4	-	23.9 ± 0.1	19.3 ± 0.5	5.0 ± 0.2
	-/-	**31.0 ± 0.4**	-	**17.7 ± 0.1**	**15.8 ± 0.4**	**9.5 ± 0.1**
Kidney	+/+	23.7 ± 0.4	6.2 ± 0.4	9.6 ± 0.6	37.7 ± 1.0	5.3 ± 0.1
	-/-	**32.6 ± 0.4**	**19.1 ± 1.3**	**5.3 ± 0.4**	**14.0 ± 0.3**	**12.2 ± 0.4**
Spleen	+/+	27.5 ± 0.6	7.5 ± 0.9	10.0 ± 0.5	35.6 ± 1.4	6.7 ± 0.3
	-/-	**36.4 ± 0.9**	**20.9 ± 1.4**	**5.1 ± 0.5**	**14.5 ± 0.6**	**14.3 ± 0.6**
Heart	+/+	15.0 ± 1.5	3.3 ± 1.0	15.3 ± 1.4	31.5 ± 2.8	6.2 ± 0.3
	-/-	24.1 ± 2.4	12.3 ± 2.7	**8.6 ± 1.2**	**16.3 ± 0.5**	**17.5 ± 1.3**
Lung	+/+	33.5 ± 1.0	8.2 ± 0.5	8.6 ± 0.7	37.9 ± 2.1	4.8 ± 0.1
	-/-	**43.3 ± 0.3**	**21.2 ± 1.1**	**4.7 ± 0.5**	**13.2 ± 0.2**	**11.4 ± 0.2**
Mammary gland	+/+	21.9 ± 0.2	9.0 ± 0.4	4.5 ± 0.3	55.8 ± 0.5	5.2 ± 0.1
	-/-	**32.7 ± 1.0**	**28.1 ± 0.8**	**0.6 ± 0.0**	**19.7 ± 0.7**	**13.6 ± 0.5**
Small Int	+/+	22.5 ± 0.3	5.6 ± 0.7	13.2 ± 1.5	38.7 ± 3.3	5.2 ± 0.2
	-/-	**32.9 ± 0.7**	**18.1 ± 1.6**	8.1 ± 0.8	**15.4 ± 0.4**	**14.4 ± 0.4**
Adrenal gland	+/+	23.7	10.3	5.2	42.4	8.2
	-/-	34.0	20.5	3.0	15.7	18.0
Testis	+/+	30.0 ± 2.4	8.0 ± 1.5	6.3 ± 0.8	35.3 ± 3.6	7.2 ± 0.4
	-/-	35.1 ± 1.7	18.8 ± 3.4	5.1 ± 0.7	**16.6 ± 0.4**	**13.3 ± 0.9**
Skin	+/+	18.0 ± 0.5	15.1 ± 0.4	1.7 ± 0.2	51.1 ± 0.9	8.8 ± 0.4
	-/-	**27.9 ± 1.2**	**31.5 ± 1.2**	1.1 ± 0.2	**20.2 ± 0.8**	**14.7 ± 0.8**
Ovary	+/+	24.3	9.5	4.1	52.4	5.8
	-/-	31.2	32.4	1.1	17.0	13.6
Eye	+/+	22.8	3.3	18.2	23.3	5.5
	-/-	32.5	11.8	11.4	13.0	11.2

Male and female mice were fed a fat free/high carbohydrate diet for 10 weeks. Fatty acids from total lipids were extracted and methylesterified and separated by GLC. Values are the mean ± S.E. of 5 mice. Bold values represent statistical significance of p<0.01 (Student's *t*-test)

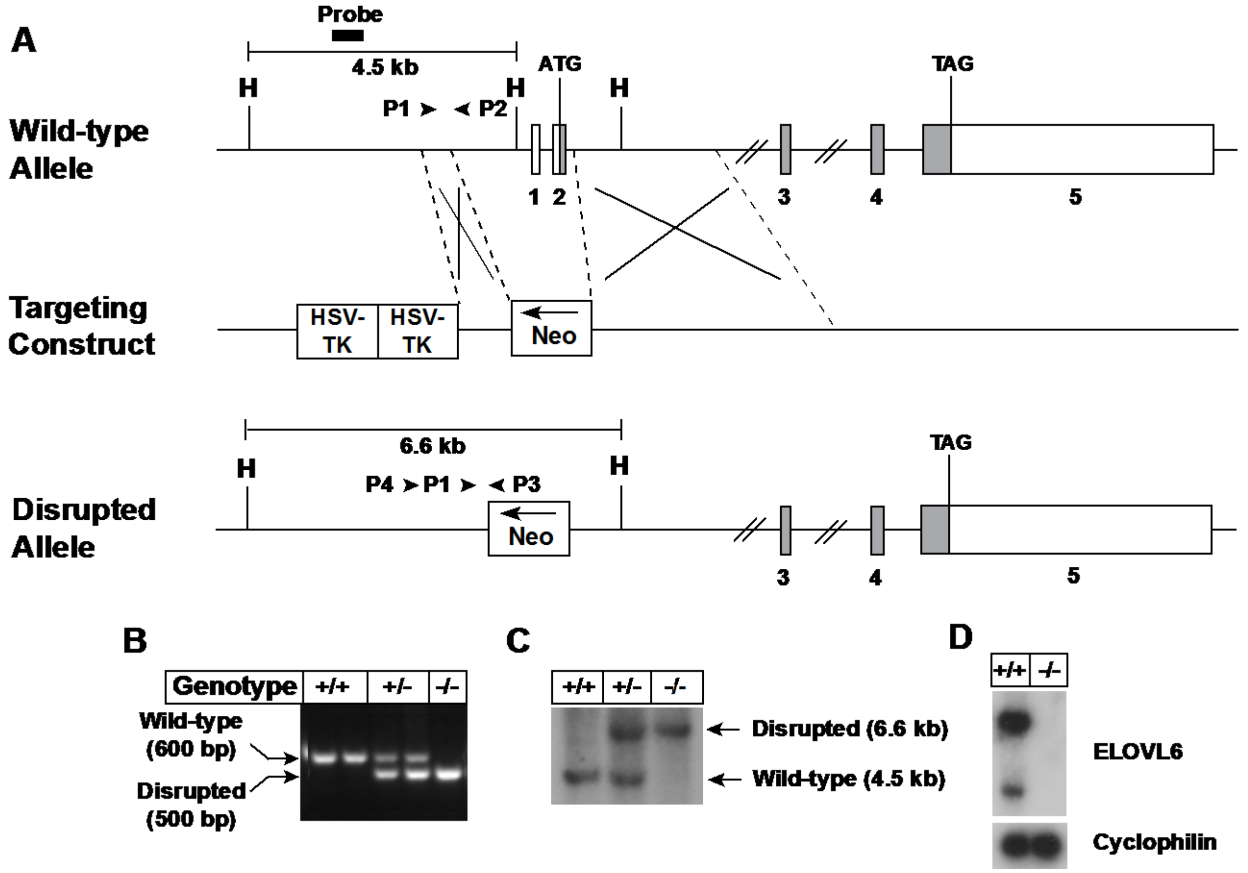

Fig. S1. Strategy for targeted disruption of the *Elovl6*. (A) Schematic of gene-targeting strategy. The gene-replacement vector was constructed as described in "EXPERIMENTAL PROCEDURES." The transcriptional direction of neo gene is shown by the arrow in the box. *Hind*III restriction sites and the expected DNA bands produced by *Hind*III digestion are shown. The location of the probe for Southern blotting is shown by a filled box. The position of primers used for ES cell screening (P4 and P3) and genotyping (P1, P2, and P3) are denoted by arrowheads. (B) Genotyping results of wild-type (+/+), heterozygote (+/-), and *Elovl6*-knockout (-/-) by PCR using P1, P2, and P3 primers. (C) Southern blot results of *Hind*III digested genomic DNA from wild-type (+/+), heterozygote (+/-), and *Elovl6*$^{-/-}$ (-/-) mice. (D) Total RNA prepared from wild-type (+/+) and *Elovl6*$^{-/-}$ (-/-) mice was subjected to northern blot analysis using a full-length ^{32}P-labeled *Elovl6* cDNA and cyclophilin as the loading control.

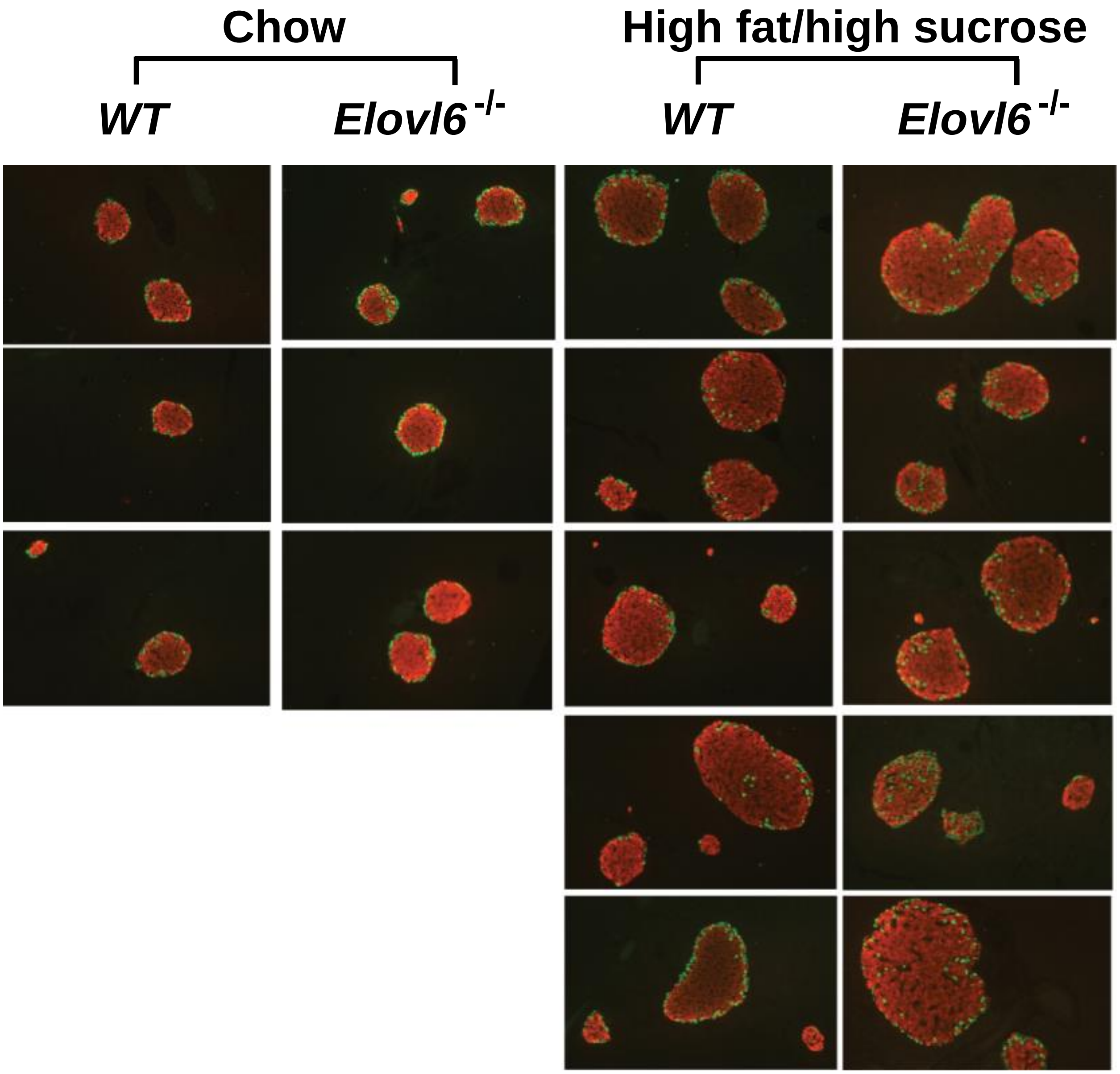

Fig. S2. Representative immunofluorescent staining of pancreatic islets from wild-type and *Elovl6*$^{-/-}$ mice. Pancreas was taken from wild-type and Elovl6-/- mice fed a chow or a high fat/high sucrose diet for 9 weeks. Immunoflorescence from insulin (red) and glucagon (green) are shown (magnification 50×).

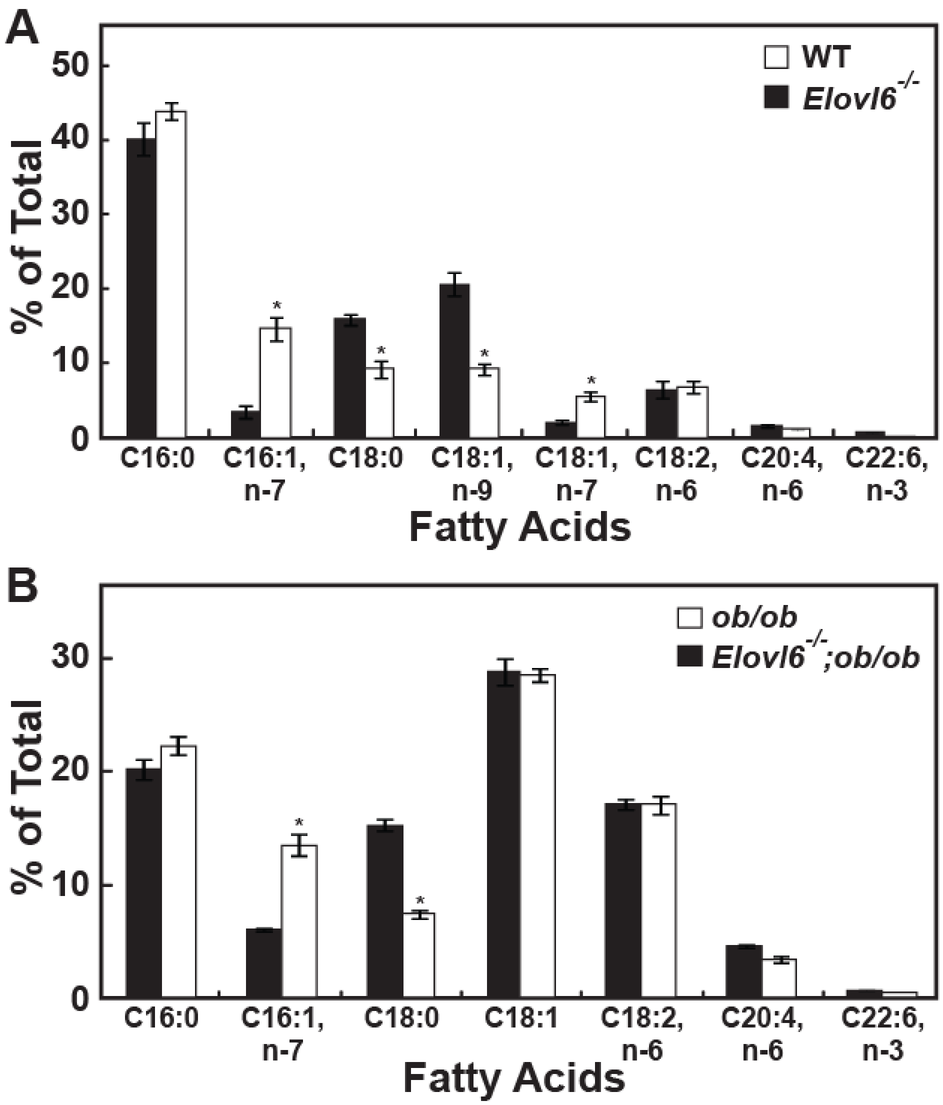

Fig. S3. Fatty acid compositions of plasma free fatty acids of wild-type and *Elovl6*$^{-/-}$ mice fed a fat free/high carbohydrate diet and of *ob/ob* and *Elovl6*$^{-/-}$*;ob/ob* mice. Free fatty acids were extracted from plasma of wild-type and *Elovl6*$^{-/-}$ mice fed a fat free/ high carbohydrate diet (A) or plasma of *ob/ob* and *Elovl6*$^{-/-}$*;ob/ob* mice (B). Relative amounts of the indicated fatty acids compared to the total fatty acids were determined. The values are the mean ± S.E. of 5 mice. * indicates $p < 0.05$ of Student *t*-test by comparison of wild-type and *Elovl6*$^{-/-}$ or *ob/ob* and *Elovl6*$^{-/-}$*;ob/ob* mice.
